# Prostaglandin E_2_ (PGE_2_) Exerts Biphasic Effects on Human Tendon Stem Cells

**DOI:** 10.1371/journal.pone.0087706

**Published:** 2014-02-04

**Authors:** Jianying Zhang, James H-C. Wang

**Affiliations:** MechanoBiology Laboratory, Departments of Orthopaedic Surgery, Bioengineering, Mechanical Engineering and Materials Science, and Physical Medicine and Rehabilitation, University of Pittsburgh, Pittsburgh, Pennsylvania, United States of America; University of Torino, Italy

## Abstract

Prostaglandin E_2_ (PGE_2_) has been reported to exert different effects on tissues at low and high levels. In the present study, cell culture experiments were performed to determine the potential biphasic effects of PGE_2_ on human tendon stem/progenitor cells (hTSCs). After treatment with PGE_2_, hTSC proliferation, stemness, and differentiation were analyzed. We found that high concentrations of PGE_2_ (>1 ng/ml) decreased cell proliferation and induced non-tenocyte differentiation. However, at lower concentrations (<1 ng/ml), PGE_2_ markedly enhanced hTSC proliferation. The expression levels of stem cell marker genes, specifically SSEA-4 and Stro-1, were more extensive in hTSCs treated with low concentrations of PGE_2_ than in cells treated with high levels of PGE_2_. Moreover, high levels of PGE_2_ induced hTSCs to differentiate aberrantly into non-tenocytes, which was evident by the high levels of PPARγ, collagen type II, and osteocalcin expression in hTSCs treated with PGE_2_ at concentrations >1 ng/ml. The findings of this study reveal that PGE_2_ can exhibit biphasic effects on hTSCs, indicating that while high PGE_2_ concentrations may be detrimental to tendons, low levels of PGE_2_ may play a vital role in the maintenance of tendon homeostasis *in vivo*.

## Introduction

Tendons transmit muscular forces to bone and, as a result, they are subjected to large mechanical loads *in vivo*. Consequently, tendons are frequently injured, especially during intense sport activities. Tendon injuries are generally difficult to treat; tendinopathy, a chronic tendon disorder involving tendon inflammation and/or degeneration, is a particularly significant challenge in orthopaedics and sports medicine. Thus far, strategies that stimulate the complete regeneration of tendons after injury have not been developed. To this end, a better understanding of tendon cell biology is essential to devise improved treatment options for tendon injuries such as tendinopathy [1].

One of the major causative factors that contribute to the development of tendinopathy is excessive mechanical loading (or overuse and over-loading) placed on tendons [2], [3]. Such excessive mechanical loading has been shown to increase the production of prostaglandin E_2_ (PGE_2_) in cultures of human tendon fibroblasts (tenocytes) *in vitro* [3], [4]. In addition, PGE_2_ production was shown to increase after exercise in the peritendinous space of Achilles tendons *in vivo* [5].

Although PGE_2_ levels increase after mechanical loading, baseline levels of PGE_2_ are present in the patellar and Achilles tendons of mice under normal conditions without mechanical loading such as treadmill running [6]. This suggests that PGE_2_ could have an impact on the tendon stem/progenitor cells (TSCs) that reside in tendons [6]–[8] and could play an important physiological role in the maintenance of tendon homeostasis. Therefore, PGE_2_ may have biphasic effects depending on its concentration. A better understanding of the concentration-dependent effects of PGE_2_ on tendon cells, particularly TSCs, may shed new light on tendon physiology and pathology. Thus, in this study we hypothesized that lower concentrations of PGE_2_ increase TSC proliferation and decrease non-tenocyte differentiation of TSCs, while higher concentrations produce the opposite effects. To test this hypothesis, we carried out cell culture experiments by treating human TSCs (hTSCs) with low and high levels of PGE_2_. We also performed *in vivo* implantation experiments to determine the differentiation fate of hTSCs after treatment with various concentrations of PGE_2_ *in vitro.*

## Materials and Methods

### Ethics Statement

The Gift of Hope Organ and Tissue Donor Network (Elmhurst, IL) provided normal human knee tissues, after obtaining written consent from donors’ families and approval from the local ethics committee (Gift of Hope Organ and Tissue Donor Network). The University of Pittsburgh IRB also approved the study protocol for using human tendon tissues in the cell culture and animal studies performed in this study. These specimens were used for investigation only and no human subjects were involved in this project. Data obtained for the study was not through intervention or interaction with individuals and does not have any identifiable private information. Further, the University of Pittsburgh IACUC approved the protocol for the use of rats in the *in vivo* implantation experiments.

### hTSC Culture

hTSCs were isolated from the patellar tendons of six human donors (20 to 44 years old) using our previously published protocol [8]. Briefly, after removing the paratenons, the core portions of the patellar tendons were cut into small pieces and digested with collagenase type I (3 mg/ml) and dispase (4 mg/ml) at 37°C for 1 hr. After centrifugation at 3,000 rpm for 15 min and removal of the enzyme-containing supernatant, a single-cell suspension was obtained, which was cultured in growth medium (DMEM plus 20% FBS) at 37°C with 5% CO_2_. After 8 to 10 days in culture dishes, hTSCs formed colonies. The stem cell colonies were then isolated and cultured in DMEM with 20% FBS. These hTSCs at passage 1 were used in the following experiments.

### Verification of the Stemness of hTSCs

The stemness of human tendon stem cells (hTSCs) from the patellar tendon used in this study was verified by immunocytochemical analysis of three stem cell markers, including octamer-binding transcription factor 4 (Oct-4), Nanog, and nucleostemin (NS). hTSCs were first seeded into 12-well plates at a density of 20,000 cells/well with 1.5 ml medium and cultured for 3 days. Then, the hTSCs were fixed in 4% paraformaldehyde in PBS for 20 min at room temperature and washed in 0.5% Triton-X-100 in PBS for 15 min. Subsequently, the fixed cells were incubated with either mouse anti-human Oct-4 (1∶500), rabbit anti-human Nanog (1∶500), or goat anti-human nucleostemin (1∶500) overnight at 4°C. After washing three times with PBS, the cells were again incubated for 2 hrs at room temperature with either Cy3-conjugated goat anti-mouse IgG antibodies (1∶1000) for Oct-4, Cy3-conjugated goat anti-rabbit IgG (1∶500) for Nanog, or Cy3-conjugated donkey anti-goat IgG antibodies (1∶500) for Nucleostemin. Nuclei were stained with Hoechst fluorochrome 33342 (1 µg/ml; Sigma, St. Louis, MO). Stained cells were then examined using fluorescence microscopy. All antibodies were obtained from Chemicon International (Temecula, CA), BD Biosciences (Franklin Lakes, NJ), Neuromics (Edina, MN), or Santa Cruz Biotechnology Inc. (Santa Cruz, CA).

### Measurement of Proliferation of hTSCs Treated with PGE_2_

hTSCs were seeded in 6-well plates (6×10^4^/well) and six different concentrations of PGE_2_ (0, 0.01, 0.1, 1, 10, and 100 ng/ml) were added to the culture. Three replicates were maintained for each concentration. The medium was changed every day and PGE_2_ was replenished. After 6 days, cell number was measured using a digital cellometer (Nexcelcom Bioscience, Lawrence, MA), and the population doubling time (PDT), which is a measure of cell proliferation, was calculated based on the formula: log_2_[Nc/N_0_], where Nc is the total number of cells at confluence, and N_0_ is the initial number of cells seeded [8].

### Determination of the Effect of PGE_2_ Treatment on hTSC Stemness

Stemness of hTSCs was determined by immunocytochemistry and FACS analysis. For immunocytochemistry, hTSCs were seeded in 12-well plates (3×10^4^/well) and treated with six different PGE_2_ concentrations ranging from 0 to 100 ng/ml for 5 days, with three replicates for each concentration. The effect of PGE_2_ treatment on hTSC stemness was then determined by performing immunocytochemistry for stem cell markers SSEA-4 and Stro-1. Briefly, cells were fixed in 4% paraformaldehyde in PBS for 30 min at room temperature. After washing with PBS, the cells were incubated at room temperature with mouse anti-human SSEA-4 (1∶350; Invitrogen, Cat. # 414000) for 3 hrs or mouse anti-human Stro-1 (1∶200; Invitrogen, Cat. # 398401) for 4 hrs. The cells were then washed three times with PBS, followed by incubation with Cy3-conjugated goat anti-mouse IgG (1∶500; Invitrogen, Cat. # A10521) secondary antibody at room temperature for 2 hrs. After a final wash with PBS, the nuclei were stained with Hoechst fluorochrome 33342, as described above. Stained cells were examined and images of cells were obtained using a fluorescence microscope (Nikon eclipse microscope, TE2000-U).

### Semi-quantification of Positively-stained hTSCs

For the semi-quantification of stem cell markers *in vitro*, seven random images were captured from each well at a magnification of 20x under the Nikon eclipse microscope. The positively-stained cells in each picture were manually identified and analyzed using SPOT™ imaging software (Diagnostic Instruments, Inc., Sterling Heights, MI). The positive staining percentage was calculated by dividing the number of positively-stained cells by the total number of cells under the microscopic field. The average value of all seven images from each well represented the percentage of positive staining, which indicates the stemness of hTSCs in the respective PGE_2_ concentrations.

### Fluorescence Activated Cell Sorting (FACS) Analysis of hTSCs

To determine the effect of PGE_2_ treatment on hTSC stemness by FACS analysis, hTSCs (1.5×10^6^ in 50 µl PBS) were incubated with 20 µl of the appropriate serum in a centrifuge tube at 4°C for 30 min. Subsequently, 0.4 µg of mouse anti-human SSEA-4 (Cell Signaling, Cat. #4755S) or mouse anti-human Stro-1 (Millipore, Cat. #MAB4315) primary antibody was added and incubated at 4°C overnight. The cells were then washed three times with 2% FBS-PBS, followed by centrifugation at 500 g for 5 min/each time. Then the cells were treated with 1 µg Cy3 conjugated goat anti-mouse IgG secondary antibody at room temperature for 2 hrs. The cells treated with the second antibody only were used as a staining negative control. Finally, the cells were washed twice with PBS and fixed in 1% paraformaldehyde, followed by FACS analysis on a BD LSR II Flow Cytometer (BD Biosciences).

### Determination of hTSC Differentiation *in vitro* by qRT-PCR

To determine the effect of PGE_2_ treatment on the differentiation of hTSCs, we performed quantitative RT-PCR (qRT-PCR) to measure gene expression using a QIAGEN QuantiTect SYBR Green PCR Kit (QIAGEN). Briefly, total RNA was isolated from hTSCs using the RNeasy Mini Kit with an on-column DNase I digest (Qiagen, Valencia, CA). Then first-strand cDNA was reverse transcribed using SuperScript II (Invitrogen, Grand Island, NY) in a 20 µl reaction containing 1 µg total RNA. Conditions for the cDNA synthesis included 65°C for 5 min, 4°C for 1 min, 42°C for 50 min, and finally 72°C for 15 min. qRT-PCR was performed in a 25 µl PCR reaction mixture with 2 µl cDNA (∼100 ng RNA) in a Chromo 4 Detector (MJ Research, Maltham, MA) by incubating at 94°C for 5 min, followed by 30 to 60 cycles of a three temperature program consisting of 1 min at 94°C, 40 sec at 57°C, and 40 sec at 72°C. The PCR reaction was terminated after a 10 min extension at 70°C and stored at 4°C until further analysis. Expression of stem cell markers (Oct-4 and Nanog), tenocyte markers (collagen type I and tenascin C), adipocyte marker (PPARγ), chondrocyte marker (Sox9), and osteocyte marker (Runx2) were measured using the primers listed in **Table 1**. GAPDH was used as an internal control. All primers were synthesized by Invitrogen. Expression of each target gene was normalized to GAPDH gene expression and the relative gene expression levels were calculated from the formula 2^−ΔΔCT^. Details of the calculation are described in our previous study [9]. The mean and standard deviation (SD) of the CT values were determined from at least three replicates.

**Table 1:** Primers used in qRT-PCR for gene expression analysis.

Gene	Primer Sequence	Accession numbers	Reference
Oct-4	Forward 5′-CGC AAG CCC TCA TTT CAC-3′	NM_002701	[32]
	Reverse 5′-CAT CAC CTC CAC CAC CTG-3′		
Nanog	Forward 5′-TCC TCC TCT TCC TCT ATA CTA AC-3′	NM_024865	[33]
	Reverse 5′-CCC ACA ATC ACA GGC ATA C-3′		
Tenascin C	Forward 5′- CGG GGC TAT AGA ACA CCA GT-3′	NM_002160.2	[34]
	Reverse 5′- AAC ATT TAA GTT TCC AAT TTC AGG TT-3′		
Collagen I	Forward 5′-AGG GTG AGA CAG GCG AAC AG-3′	NM_000088	[35]
	Reverse 5′-CTC TTG AGG TGG CTG GGG CA-3′		
PPARγ	Forward 5′- GGC TTC ATG ACA AGG GAG TTT C-3′	NM_138711	[36]
	Reverse 5′- CTT TAT GGA GCC CAA GTT TGA GTT-3′		
Sox9	Forward 5′- CCC CAA CAG ATC GCC TAC AG-3′	NM_000346	[37]
	Reverse 5′- GAG TTC TGG TCG GTG TAG TC-3′		
Runx2	Forward 5′- ATG CTT CAT TCG CCT CAC AAA-3′	NM_001015051	[38]
	Reverse 5′- CCA AAA GAA GTT TTG CTG ACA TGG-3′		
GAPDH	Forward 5′-GCC AAA AGG GTC ATC ATC-3′	NM_002046	[32]
	Reverse 5′-ATG ACC TTG CCC ACA GCC TT-3′		

### Determination of hTSC Differentiation *in vivo* by Implantation

To verify the effect of PGE_2_ treatment on the differentiation of hTSCs *in vivo*, eight female nude rats (10 weeks old; 200–250 g) were used. hTSCs at passage 2 were seeded into 24-well plates (8×10^6^ cells/well) and cultured in DMEM with or without various concentrations of PGE_2_ for 6 days, with a change of medium every day. For implantation experiments, the cells were trypsinized from each well and mixed with 0.25 ml Matrigel (BD Scientific) to enable gel formation after implantation. These hTSC-Matrigel mixtures were placed subcutaneously in the back of anesthetized rats. Three pieces of hTSC-Matrigel were positioned in three distinct places on each side of each rat’s back. Four weeks after implantation, tissue samples were harvested from the implanted area and placed in pre-labeled base molds filled with frozen section medium (Neg 50; Richard-Allan Scientific; Kalamazoo, MI). The tissue blocks were stored at −80°C until histological analysis.

### Immunohistochemical and Histological Analyses

Each frozen tissue block was cut into 10 µm thick sections, placed on glass slides, and then allowed to dry overnight at room temperature. The tissue sections were fixed in 4% paraformaldehyde for 30 min and further washed three times with PBS. They were then incubated at room temperature with mouse anti-human PPARγ antibody (Santa Cruz Biotechnology, Inc., Cat. #271392, Santa Cruz, CA) diluted to 1∶350 for 2 hrs, mouse anti-collagen type II antibody (1∶300; Millipore, Cat. #MAB8887, Temecula, CA) for 2 hrs, or mouse anti-human osteocalcin antibody (1∶300; Santa Cruz Biotechnology, Inc., Cat. #74495, Santa Cruz, CA) for 3 hrs. After washing with PBS, Cy3-conjugated goat anti-mouse IgG (1∶500; Santa Cruz Biotechnology) was added as secondary antibody and incubated at room temperature for 1 hr, followed by staining the nuclei with Hoechst fluorochrome 33342 (1 µg/ml; Sigma, St. Louis, MO) at room temperature for 5 min. Additionally, cell morphology and distribution in those tissues that received hTSCs, which had been treated with various concentrations of PGE_2_ in culture, were assessed by staining with hematoxylin and eosin (H&E). Finally, all tissue sections were examined under a fluorescence microscope.

### Semi-quantification of Positively Stained Tissue Sections

Each tissue section was examined under a microscope (Nikon eclipse, TE2000-U) and five random images were taken for the semi-quantification of hTSC differentiation *in vivo*. SPOT™ imaging software (Diagnostic Instruments, Inc., Sterling Heights, MI) was used to process positively stained areas, which were manually identified by examining the images taken. The total area viewed under the microscope was divided by the positively stained area to calculate the proportion of positive staining. Five tissue sections were used for each group and five images were obtained per tissue section. These values were averaged to represent the percentage positive staining in all the groups treated with various PGE_2_ concentrations, which indicated the extent of cell differentiation.

### Statistical Analysis

Data are expressed as mean ± standard deviation (mean ± SD). Unless otherwise indicated, at least three replicates were used for each experimental condition. For statistical analysis of data, one-way ANOVA or a student *t*-test was used wherever appropriate. All comparisons were between each PGE_2_-treated group and the respective control. A P-value less than 0.05 was considered to indicate statistically-significant differences between the groups compared.

## Results

### Verification of the Stemness of hTSCs

**Figure 1:**
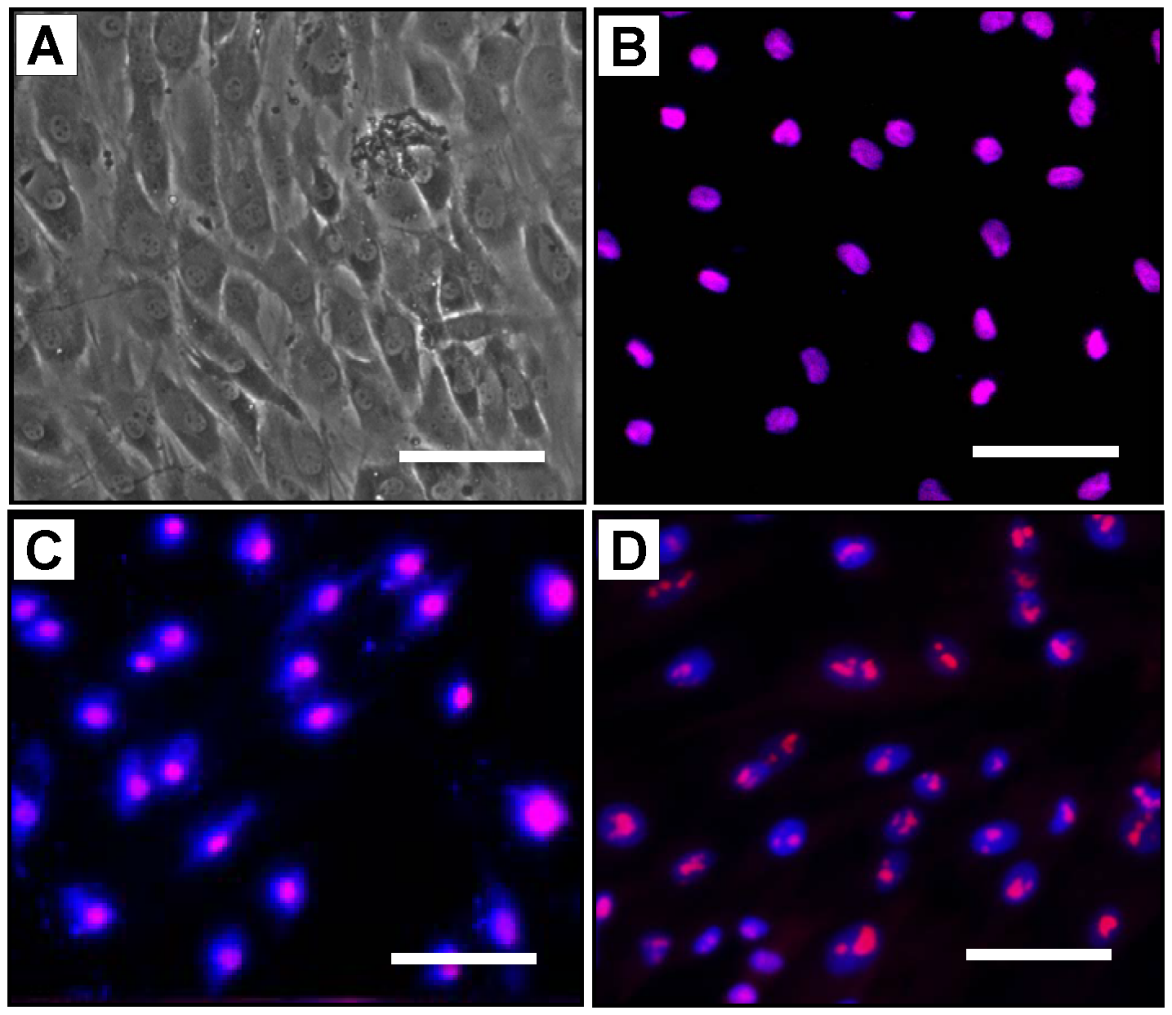
Verification of the stemness of hTSCs. Cobblestone shaped morphology of hTSCs visualized under phase contrast microcopy (**A**). hTSCs also expressed Oct-4 (**B**), Nanog (**C**), and nucleostemin (**D**). Staining for all three stem cell markers was nearly 100% positive with the respective antibodies. Bar = 50 µm.

Prior to using hTSCs for cell culture experiments in this study, we first verified the stemness of these tendon cells. Microscopic examination of hTSCs revealed the typical cobblestone-shaped morphology of tendon stem cells under phase contrast microcopy (**Fig. 1A**). Further, cells in culture also showed robust expression of all three stem cells markers, Oct-4 (**Fig. 1B** **)**, Nanog (**Fig. 1C**), and nucleostemin (**Fig.** **1D**), in immunohistochemical analyses. These characteristics indicated that the cells derived from the human patellar tendons were indeed tendon-specific stem cells.

### Effect of PGE_2_ on the Proliferation of hTSCs

**Figure 2:**
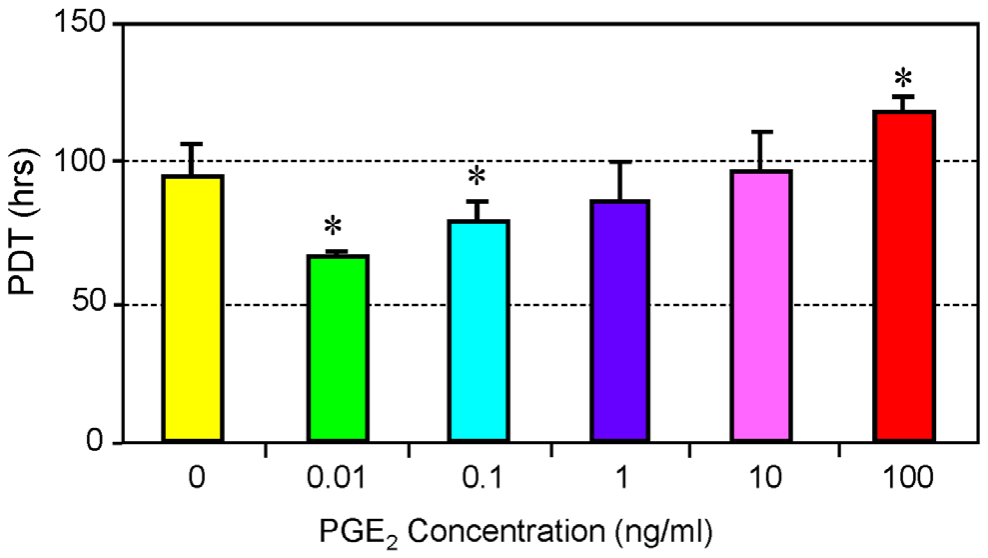
Population doubling time (PDT) of hTSCs treated with various concentrations of PGE_2_. hTSCs were seeded in 6-well plates and cultured for six days on medium containing six different concentrations of PGE_2_. PDT increased with increasing concentration of PGE_2_, meaning that increased PGE_2_ resulted in decreased cell proliferation (*p<0.05 when compared to control cells without PGE_2_ treatment).

After establishing that the cells in culture were hTSCs, we investigated cell proliferation after PGE_2_ treatment of hTSCs by determining their population doubling time (PDT). Treatment of hTSCs with a lower concentration (0.01 ng/ml) of PGE_2_ significantly increased cell proliferation, as evidenced by decreased PDT when compared to the control (**Fig. 2**). PGE_2_ treatment at a higher concentration (0.1 ng/ml) also induced similar proliferative effects, although to a smaller extent. At concentrations of 1 and 10 ng/ml, the proliferation of hTSCs was not significantly different from the control without PGE_2_ treatment. At the highest concentration (100 ng/ml), TSC proliferation was significantly decreased.

### Effect of PGE_2_ Treatment on the Stemness of hTSCs

**Figure 3:**
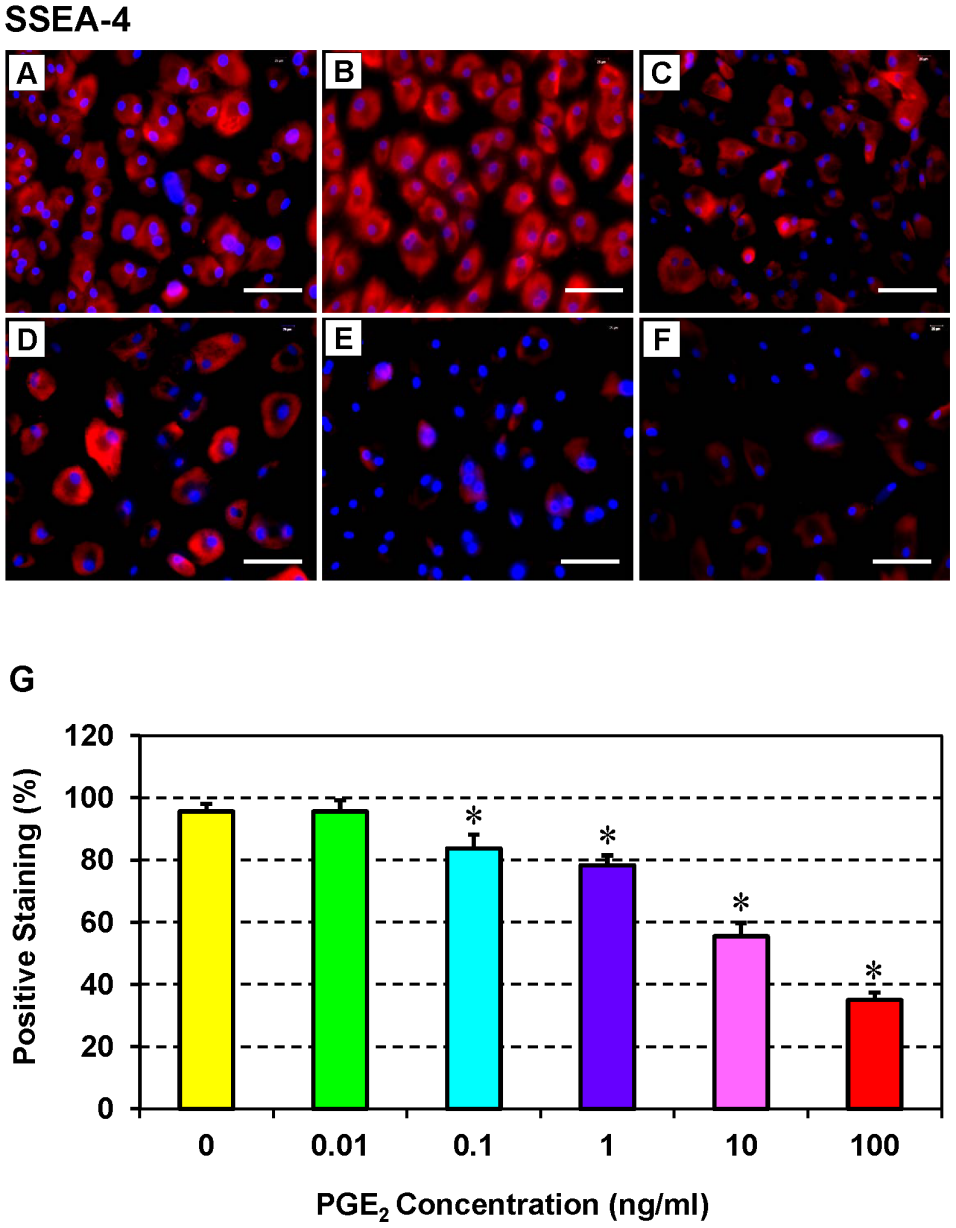
Expression of the stem cell marker SSEA-4 by hTSCs cultured *in vitro* in various concentrations of PGE_2_. **A**: without PGE_2_ treatment; **B**: 0.01 ng/ml PGE_2_; **C**: 0.1 ng/ml PGE_2_; **D**: 1 ng/ml PGE_2_; **E**: 10 ng/ml PGE_2_; and **F**: 100 ng/ml PGE_2_. hTSCs were seeded in 12-well plates, cultured with six different concentrations of PGE_2,_ incubated with mouse anti-human SSEA-4 primary antibody, and detected with Cy3-conjugated goat anti-mouse IgG. Nuclei were stained with Hoechst (Blue). Expression of SSEA-4 (red) is dose-dependent, with more robust expression seen in hTSCs treated with low levels of PGE_2_ (**A–D**) than expression levels seen in those treated with high levels (**E, F**). Positively stained cells were also counted to calculate percentage staining (**G**) (*p<0.05 with respect to hTSCs not treated with PGE_2_). Bar: 100 µm.

**Figure 4:**
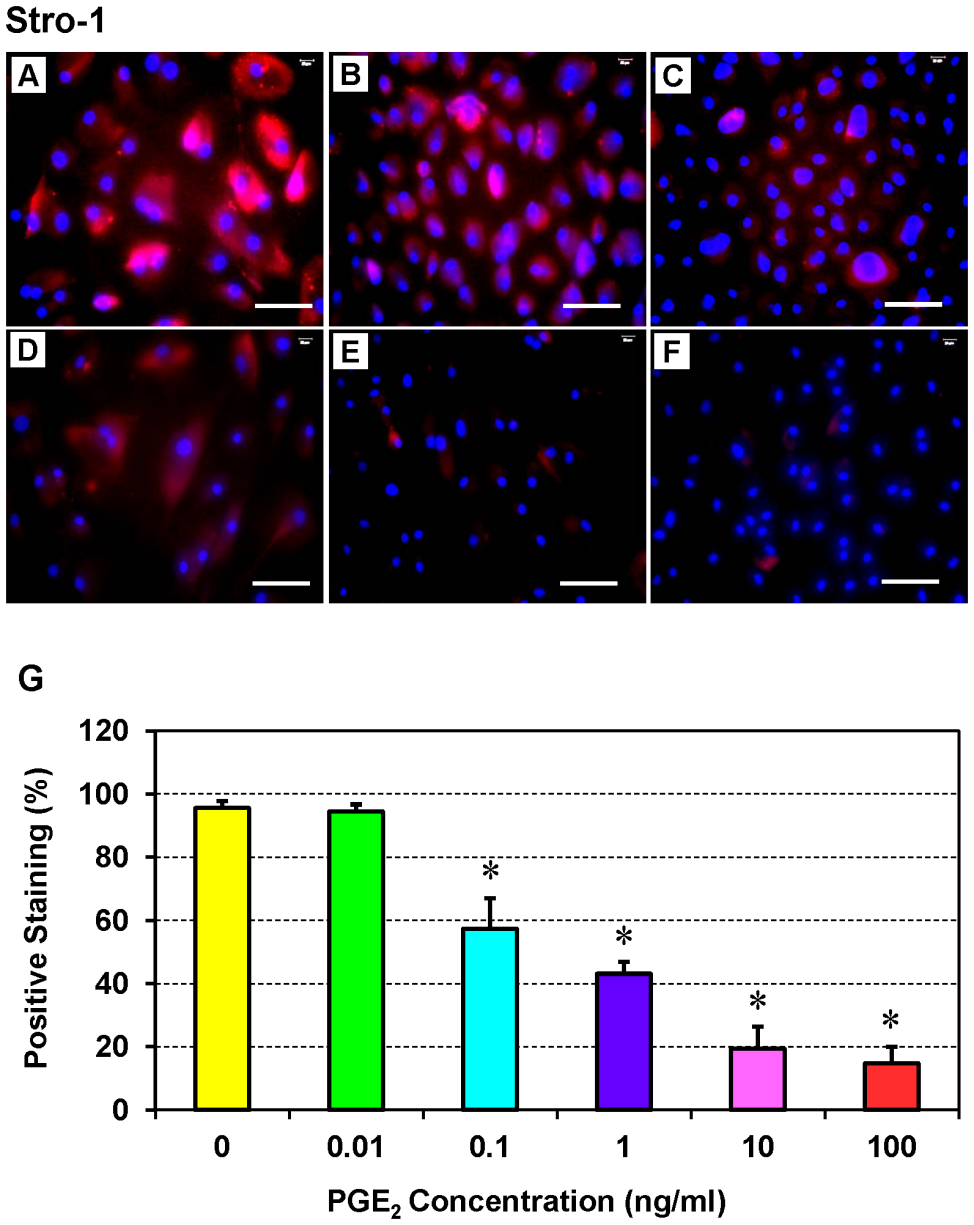
Expression of the stem cell marker Stro-1 by hTSCs cultured *in vitro* in medium containing various concentrations of PGE_2_. **A**: without PGE_2_ treatment; **B**: 0.01 ng/ml PGE_2_; **C**: 0.1 ng/ml PGE_2_; **D**: 1 ng/ml PGE_2_; **E**: 10 ng/ml PGE_2_; and **F**: 100 ng/ml PGE_2_. hTSCs were seeded in 12-well plates, cultured with six different concentrations of PGE_2,_ incubated with mouse anti-human Stro-1, and detected with Cy3-conjugated goat anti-mouse IgG. Hoechst was used to stain nuclei (blue). Expression of Stro-1 (red) is higher in hTSCs treated with low PGE_2_ concentrations (**A, B)** than hTSCs treated with high concentrations (**E**–**F**). Similar to SSEA-4, expression of Stro-1 is also dose-dependent. Positively stained cells were also counted to calculate percentage staining (**G**) (*p<0.05 in comparison with control hTSCs not treated with PGE_2_). Bar: 100 µm.

Immunofluorescence assays for stem cell markers revealed that hTSCs treated with a low concentration of PGE_2_ (0.01 ng/ml) expressed SSEA-4 (**Fig. 3B**) and Stro-1 (**Fig. 4B**) more extensively than controls (without PGE_2_ treatment) (**Fig. 3A** **,** **4A**) and those treated with higher concentrations of PGE_2_ (10 or 100 ng/ml) (**Fig. 3E, 3F** **,** **4E, 4F**). Indeed, the expression levels of both stem cell markers were significantly inhibited by higher concentrations of PGE_2_ (10 or 100 ng/ml) (**Fig. 3** **,** **4**). However, semi-quantification of the staining results revealed that the levels of both SSEA-4 (**Fig. 3G**) and Stro-1 (**Fig. 4G**) were similar between the control hTSCs and hTSCs treated with 0.01 ng/ml PGE_2_. Consistent with the microscopic observations, higher concentrations of PGE_2_ significantly reduced staining for both stem cell markers. Particularly, the concentration-dependent effect of PGE_2_ on Stro-1 was more profound than its effect on SSEA-4 (**Fig. 3G** **,** **4G**), with 81% reduction at 100 ng/ml, 76% at 10 ng/ml, 52% at 1 ng/ml, and 38% at 0.1 ng/ml for Stro-1, and 61% at 100 ng/ml, 40% at 10 ng/ml, 17% at 1 ng/ml, and 12% at 0.1 ng/ml for SSEA-4.

**Figure 5:**
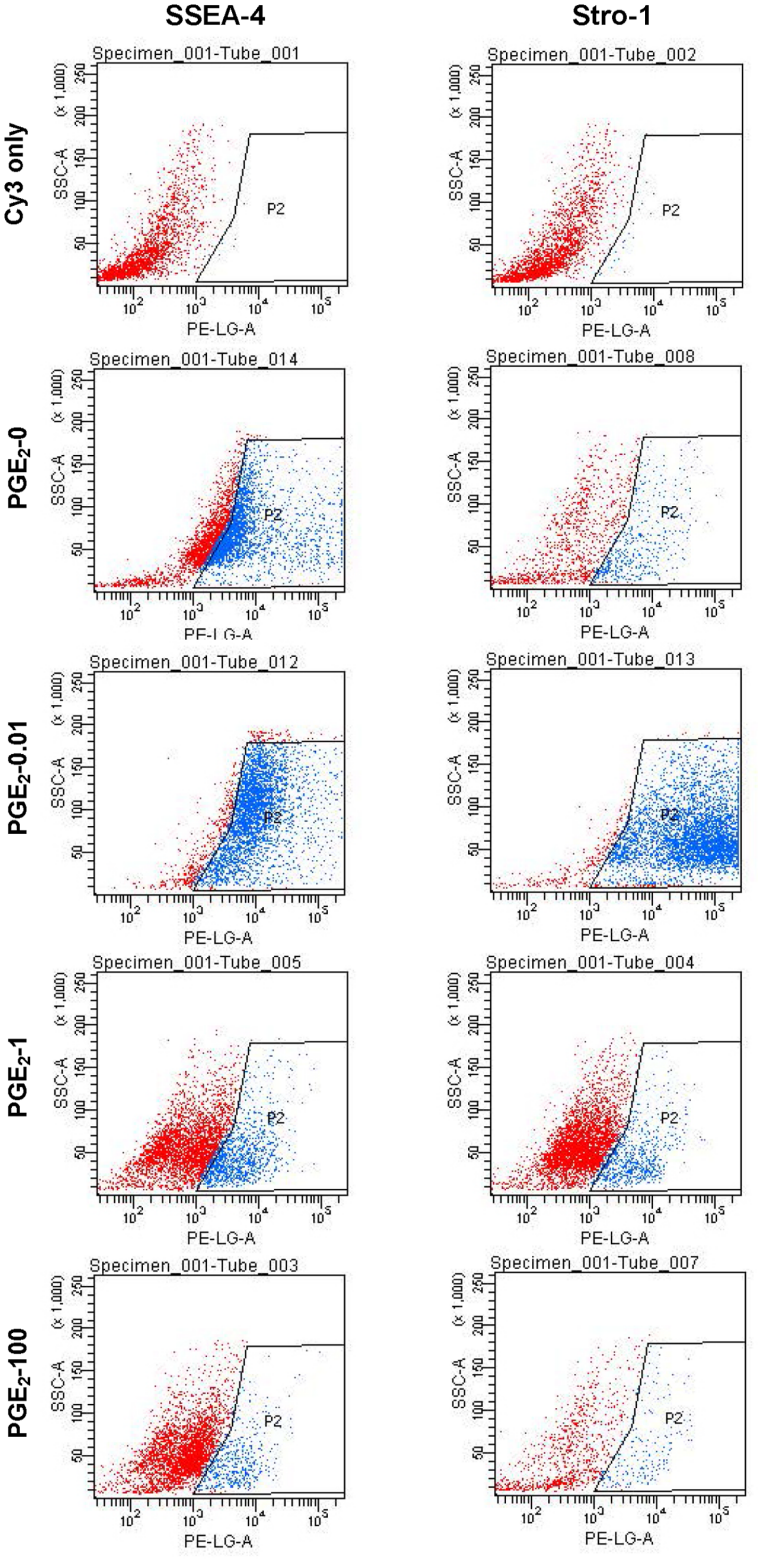
FACS analysis of SSEA-4 and Stro-1 expression in hTSCs treated with various concentrations of PGE_2_. hTSCs in culture were treated with various concentrations of PGE_2_. FACS analysis was performed on these cells (for details, see Methods section). It is evident that when cells were treated with 0.01 ng/ml of PGE_2_ (PGE_2_-0.01), more cells positively stained with SSEA-4 and Stro-1 were detected (blue dots in the P2 area) compared to control cells without PGE_2_ treatment (PGE_2_-0). When PGE_2_ concentration increased to 1 ng/ml (PGE_2_-1) and 100 ng/ml (PGE_2_-100), fewer cells were actually stained with SSEA-4 and Stro-1 when compared to control cells (PGE_2_-0).

**Figure 6:**
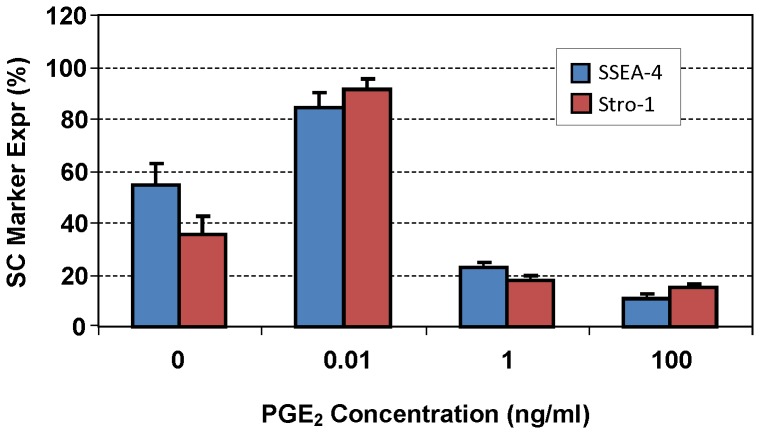
Quantification of SSEA-4 and Stro-1 expression in hTSCs treated with various concentrations of PGE_2_. Average percentage of cells expressing SSEA-4 and Stro-1 in two independent FACS experiments showed that hTSCs treated with 0.01 ng/ml of PGE_2_ expressed the most SSEA-4 (84.6±5.4%) and Stro-1 (91.1±4.2%), which was higher than the control cells without PGE_2_ treatment (54.5±8.6% for SSEA-4, and 35.8±6.5% for Stro-1) and the cells treated with 1 ng/ml (SSEA-4∶23.0±2.0%; and Stro-1∶17.7±1.7%) or 100 ng/ml PGE_2_ (SSEA-4∶10.6±2.1%, and Stro-1∶15.3±1.1%). SC: Stem cell; PGE_2_-0: PGE_2_ at 0 concentration; PGE_2_-0.01: PGE_2_ concentration at 0.01 ng/ml; PGE_2_-1: PGE_2_ concentration at 1 ng/ml; and PGE_2_-100: PGE_2_ concentration at 100 ng/ml.

Additionally, FACS analysis of the stem cell markers also corroborated the immunocytochemical findings. Specifically, as PGE_2_ concentrations increased from 0 to 0.01 ng/ml, more cells positively stained with SSEA-4 and Stro-1 (**Fig. 5**, blue dots) were evident; however, when PGE_2_ concentrations were further increased to 1 and 100 ng/ml, few positively-stained cells were detected. Quantification of the results from two independent FACS experiments also confirmed these observations (**Fig. 6**).

**Figure 7:**
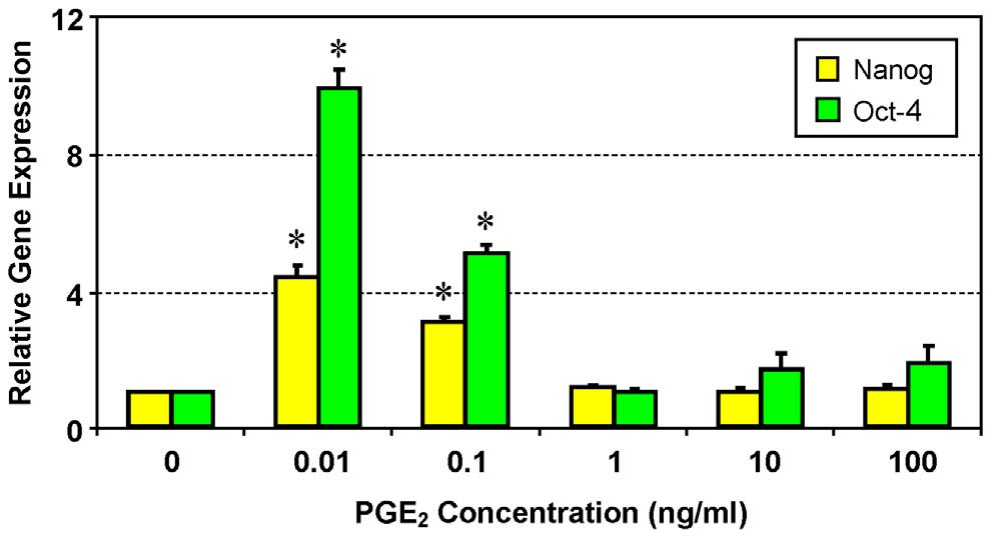
Expression of stem cell markers Nanog and Oct-4 in hTSCs treated with various concentrations of PGE_2_. Total RNA was collected from various hTSCs cultured with or without PGE_2_ and used for qRT-PCR. Expression levels of Nanog and Oct-4 are more up-regulated in hTSCs treated with low concentrations of PGE_2_ (0.01 and 0.1 ng/ml) than in those treated with high concentrations (1, 10, and 100 ng/ml) (*p<0.05 with respect to hTSCs not treated with PGE_2_).

To further characterize the stemness of hTSCs after treatment with PGE_2_, we examined the expression of stem cell genes using qRT-PCR. We found that the gene expression levels of Nanog and Oct-4 were significantly (p<0.05) up-regulated in hTSCs treated with lower concentrations (0.01 and 0.1 ng/ml) of PGE_2_ (**Fig. 7**). Notably, the expression level of Oct-4 was twice as high as that of Nanog at 0.01 ng/ml PGE_2_ concentration. When treated with higher concentrations (1, 10, and 100 ng/ml) of PGE_2_, expression levels of both Nanog and Oct-4 were down-regulated and almost reached the levels of controls without PGE_2_ treatment.

### Effect of PGE_2_ on the Differentiation of hTSCs

**Figure 8:**
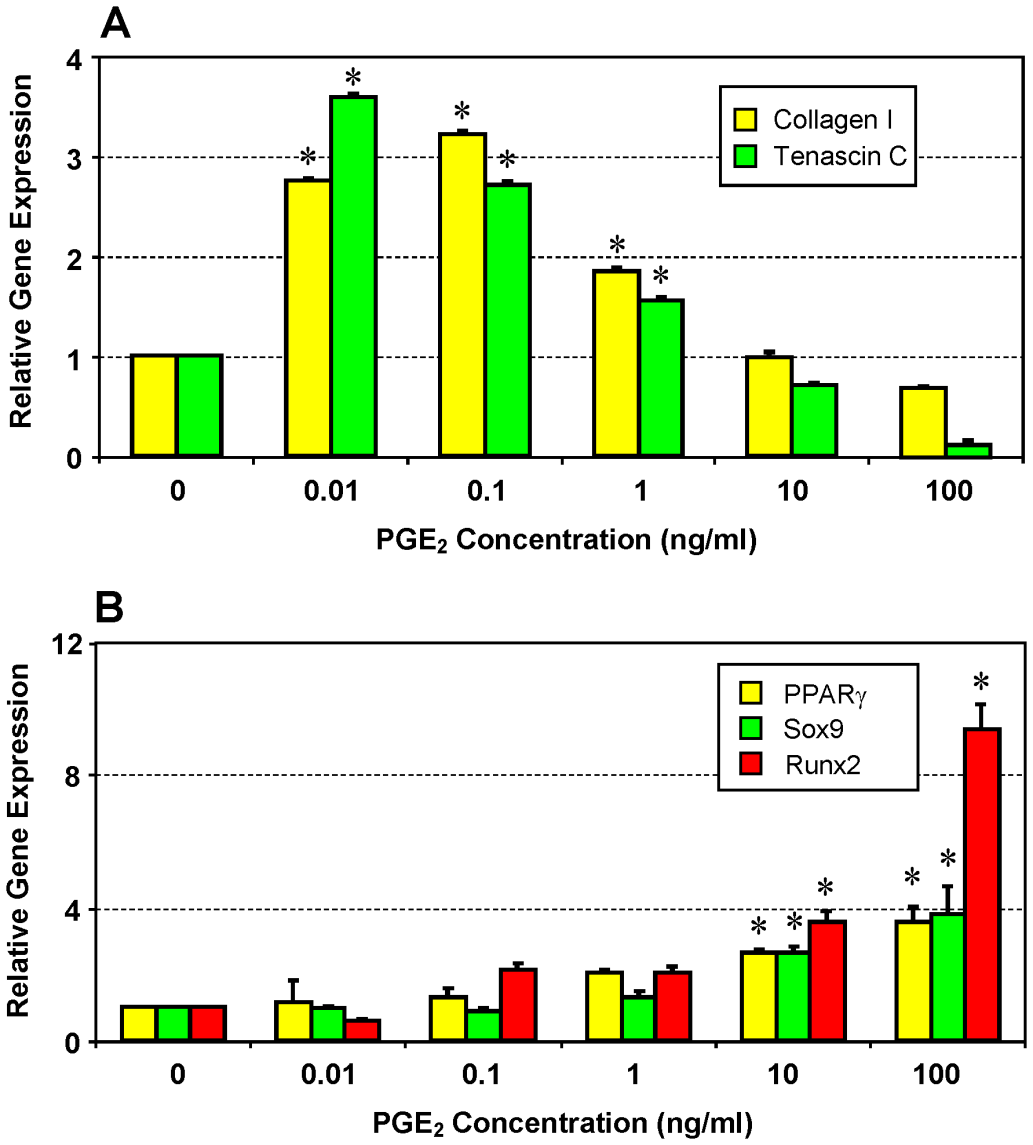
Expression of tenocyte (A) and non-tenocyte (B) related genes in hTSCs treated with various concentrations of PGE_2_. qRT-PCR was performed on total RNA collected from cultured hTSCs treated with PGE_2_. Expression levels of tenocyte related genes, collagen type I (Collagen I) and Tenascin C, were higher in hTSCs treated with low concentrations of PGE_2_ (0.01, 0.1 and 1 ng/ml) than in those treated with high concentrations (10 and 100 ng/ml) (**A**). However, expression levels of non-tenocyte related genes, PPARγ, Sox9, and Runx2, were more reduced in hTSCs treated with low (0.01, 0.1 and 1 ng/ml) than with high concentrations of PGE_2_ (10 and 100 ng/ml) (**B**) (*p<0.05 with respect to corresponding controls that did not receive PGE_2_ treatment).

We next examined the effects of PGE_2_ on hTSC differentiation by determining the expression of tenocyte and non-tenocyte related genes. Treatment of hTSCs with lower concentrations (0.01, 0.1, and 1 ng/ml) of PGE_2_ significantly (p<0.05) enhanced the expression of both collagen type I and tenascin C, two tenocyte-associated genes (**Fig. 8A**). However, at these lower concentrations, the expression levels of non-tenocyte associated genes PPARγ, Sox9, and Runx2 were lower or only marginally higher than the control (**Fig. 8B**). On the other hand, treatment of hTSCs with higher concentrations (10 and 100 ng/ml) of PGE_2_ significantly (p<0.05) up-regulated PPARγ, Sox9, and Runx2 genes associated with adipogenic, chondrogenic, and osteogenic differentiation, respectively (**Fig. 8B**). This up-regulation corresponded with the down-regulation of collagen type I and tenascin C at 10 and 100 ng/ml PGE_2_ concentrations (**Fig. 8A**).

### Non-tendinous Tissue Formation after Implantation of PGE_2_-treated hTSCs

**Figure 9:**
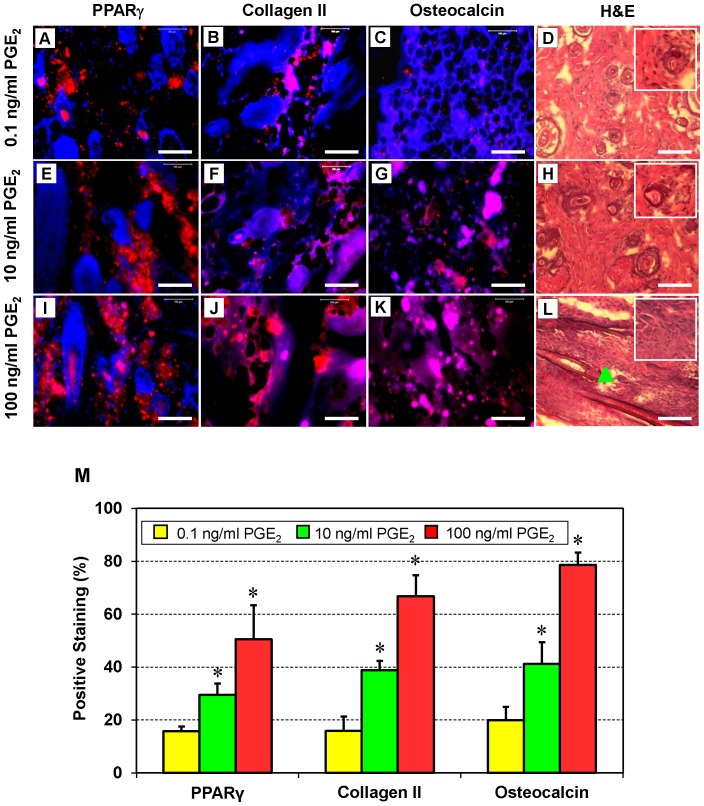
*In vivo* expression of non-tenocyte markers PPARγ, collagen type II, and osteocalcin in rats implanted with hTSCs treated with various concentrations of PGE_2_ and their respective hematoxylin and eosin (H&E) stained tissue sections. hTSCs cultured with three concentrations (0.1, 10, and 100 ng/ml) of PGE_2_ were implanted subcutaneously into rats; later, immunohistochemical and histological analyses were performed on tissue sections. For the immunohistochemical staining, fixed tissue sections were incubated with mouse anti-human PPARγ antibody, mouse anti-collagen type II (Collagen II) antibody, or mouse anti-human osteocalcin antibody. Cy3-conjugated goat anti-mouse IgG was then used to detect primary binding. Nuclei were stained with Hoechst (blue). Expression levels of PPARγ, collagen type II, and osteocalcin (red) were lower in cells treated with 0.1 ng/ml PGE_2_ (**A–C**) than those treated with the higher concentrations (10 and 100 ng/ml) of PGE_2_ (**E–G, I–K**). H&E staining was also performed on tissue sections (**D, H, L**). More cells (black dots; see insets in **D**, **H**, and **L**) were observed in tissues implanted with hTSCs that had been treated with high concentrations of PGE_2_ in culture (**H, L**). Specifically, at 100 ng/ml (**L**), cells were concentrated in a specific region (triangle). Additionally, semi-quantification of the stained cells was performed by counting immuno-positive cells and calculating percentage staining (**M**) (*p<0.05 in comparison with control hTSCs not treated with PGE_2_). Bar: 100 µm.

To determine whether PGE_2_-treated hTSCs underwent non-tenogenic differentiation, we subcutaneously implanted PGE_2_-treated hTSCs into nude rats. We found that 4 weeks after implantation, non-tenocyte differentiation of hTSCs was more extensive in the cells treated with higher concentrations (10 and 100 ng/ml) of PGE_2_ (**Fig. 9E–G** **,** and **Fig. 9I–K**) when compared to the hTSCs that received the lowest concentration of PGE_2_ (0.1 ng/ml) (**Fig. 9A–C**), as evidenced by higher amounts of PPARγ, collagen type II and osteocalcin (stained in red/pink). It appeared that more cells (black dots) were present in tissues that received hTSCs treated with high PGE_2_ concentrations (**Fig. 9H–L**) than those that were treated with low PGE_2_ concentrations (**Fig. 9D**). Specifically, at 100 ng/ml (**Fig. 9L**), numerous cells were concentrated in a specific region (triangle). The immunohistochemical observations were also confirmed by semi-quantification, which showed a significant (P<0.001) dose-dependent increase in the staining of non-tenocyte associated genes with increasing amounts of PGE_2_ (**Fig. 9M**). When compared to hTSCs treated with 0.1 ng/ml PGE_2_, those treated with 10 ng/ml had a ∼2 fold increase in PPARγ, collagen type II, and osteocalcin. These increases were higher when hTSCs were treated with 100 ng/ml PGE_2_ (∼3 fold for PPARγ, ∼4 fold for collagen type II, and ∼4 fold for osteocalcin).

## Discussion

PGE_2_ is one of the most abundant prostaglandins in the body, and an important causative factor of inflammation that results from tissue damage or infection. Since our previous study showed that high levels of PGE_2_ (1, 10, and 100 ng/ml) decrease proliferation and induce differentiation of mouse TSCs into non-tenocytes [6], in the present study we investigated the effects of comparable and lower doses of PGE_2_ (0.01 to 100 ng/ml) on hTSC proliferation and differentiation by performing cell culture and cell implantation experiments. Our results revealed a concentration-dependent biphasic effect of PGE_2_ on the proliferation and differentiation of hTSCs. PGE_2_ treatment of hTSCs increased cell proliferation at lower concentrations, but decreased it at higher concentrations. In particular, low levels of PGE_2_ promoted the stemness of hTSCs, as evidenced by the extensive expression of stem cell markers SSEA-4 and Stro-1 in hTSCs treated with low concentrations of PGE_2_. The range of PGE_2_ concentrations used in this study also includes the *in vivo* physiological concentrations of PGE_2_ reported in human Achilles tendons (0.8±0.2 ng/ml, [10] or 54±24 pg/ml [11]). It should be noted that these values are likely lower due to two reasons: i) patients in these studies were at rest during these measurements and did not undergo intensive exercise, and ii) these values are average microdialysis measurements of PGE_2_ concentrations over a large portion of the tendon instead of at a local site, where PGE_2_ concentrations could be much higher.

The biphasic effects of PGE_2_ on various tissue properties have been reported in previous studies. For example, PGE_2_ has been shown to exert biphasic effects on vascularity [12]; it elicits vasodilation at low concentrations and reverses this effect at higher concentrations. Similarly, PGE_2_ treatment reduced proliferation of mesenchymal stem cells (MSCs) in a dose-dependent manner (0.25 µM to 25 µM PGE_2_, or 88 ng/ml to 8.8 µg/ml), with the two lowest concentrations (0.25 nM and 2.5 nM PGE_2_, or 88 pg/ml to 880 pg/ml) slightly increasing MSC proliferation over baseline levels [13]. In this study, the authors demonstrated that the biphasic effect of PGE_2_ was executed by differential activation of two types of protein kinase A (PKA). At low concentrations, PGE_2_ activated PKA II, leading to a cascade of events that resulted in cell proliferation; at high concentrations, PGE_2_ caused PKA I activation, resulting in cell cycle arrest which reduced MSC proliferation. In addition, PGE_2_ was reported to have a biphasic influence on injured esophagus: at low doses PGE_2_ was protective, but at high doses it damaged the esophagus, with this effect being mediated by the EP1 receptor [14]. Interestingly, the biphasic effects of PGE_2_ were also reported to depend on the growth state of the tissue type. For example, PGE_2_ promoted proliferation of quiescent smooth muscle cells indicated by an increase in both DNA and RNA synthesis with increasing levels of PGE_2_ (10^−10^–10^−5^M, or 3.5 ng/ml - 3.5 µg/ml). However, when proliferating smooth muscle cells were treated with the same concentrations of PGE_2_, DNA synthesis decreased by 48%, indicating that PGE_2_ had an inhibitory effect [15].

In this study, we established the stemness of hTSCs based on three characteristics described previously for human tendon stem/progenitor cells: a) the ability to form colonies in culture; b) expression of stem cell markers Oct-4, Nanog, and nucleostemin; and c) multi-differentiation potential [7], [8]. In addition, these hTSCs assumed a cobblestone shape when grown to confluence [8]. Further, we used two stem cell markers, SSEA-4 and Stro-1, to measure the stemness of hTSCs treated with various concentrations of PGE_2_. SSEA-4 and Stro-1 are highly expressed in undifferentiated stem cells and therefore are used as markers for stem cell identification. However, after differentiation, SSEA-4 is down-regulated in human embryonic stem cells [16]. Our results showing higher expression of SSEA-4 in cells treated with low levels of PGE_2_ indicate that stemness is enhanced in these cells, but not in cells treated with higher levels of PGE_2_. Additionally, we also found that cells treated with low levels of PGE_2_ produced higher levels of stem cell-related genes (Oct-4 and Nanog) than cells treated with high levels of PGE_2_. Oct-4 and Nanog are both required for the self-renewal and maintenance of stem cells in an un-differentiated state [17]. These genes were reported to downregulate the expression and activity of lineage specific factors, thereby promoting pluripotency [18]. Their downregulation, however, increased differentiation and thereby decreased the capacity of mouse embryonic stem cells for self-renewal [19]–[21].

This study found that higher expression levels of both Nanog and Oct-4 and corresponded low levels of non-tenocyte related genes, particularly in cells treated with low levels of PGE_2_ (0.01, and 0.1 ng/ml). The results indicate maintenance of hTSCs in an undifferentiated state, at least in part through Nanog and Oct-4 suppression of adipocyte- (PPARγ), chondrocyte- (Sox9), and osteocyte- (Runx2) related genes. Further, lower expression levels of Nanog and Oct-4, especially in cells treated with high concentrations of PGE_2_ (10 and 100 ng/ml), also corresponded to higher expression levels of non-tenocyte related genes. This effect, however, was not observed in the control cells (those without PGE_2_ treatment), indicating the role high PGE_2_ levels have in promoting non-tenocyte differentiation of hTSCs, which in turn reduces their stemness. Taken together, these results strongly suggest that the beneficial effects of the constitutively maintained low levels of PGE_2_ may be critical for the maintenance of homeostasis in tendons *in vivo*.

hTSCs treated with higher concentrations of PGE_2_ exhibited extensive expression of non-tenocyte related genes. In the *in vivo* experiment, non-tenocyte proteins PPARγ, collagen type II, and osteocalcin were up-regulated after implantation of hTSCs treated with high levels of PGE_2_. These findings suggest that PGE_2_ at high concentrations could cause differentiation of TSCs into non-tenocytes; this could lead to impaired tendon healing and the formation of non-tendinous tissues in affected tendons, which would consequently reduce tendon strength. Indeed, it has been suggested that PGE_2_, as a local hormone in tendons, may contribute to the development of tendinopathy [2], [22]–[24]. In addition, prostaglandins (PGs) are known to play a pathophysiological role in the skeletal system, including contributing to the pathology of osteoporosis by enhancing bone resorption [25]. However, in the same milieu, PGs also exert a physiological role by stimulating bone formation through increased osteoblast proliferation and differentiation. These functions of PGs are consistent with the biphasic effects of PGE_2_ that maintain tendon homeostasis and lead to tendon pathology or tendinopathy.

It should be noted that when hTSCs were treated with low levels of PGE_2_, tenocyte-related genes, including collagen type I and tenascin C, were highly expressed (**Fig. 7A**). These results suggest that PGE_2_ at low concentrations may exert its effects on TSCs in two ways: promoting the stemness of TSCs, and inducing TSCs to differentiate towards tenocytes (or progenitor cells for tenocytes). TSCs in our cultures presumably consisted of two sub-populations of cells: one population consisted of early-stage stem cells expressing stem cell markers, such as Nanog, Oct-4, SSEA-4, and Stro-1, and the other population consisted of progenitor cells, which have differentiated towards tenocytes and expressed collagen type I and tenascin C, as demonstrated in this study. In other words, low levels of PGE_2_ not only promote TSC self-renewal, but also promote the differentiation of TSCs into progenitor cells for tenocytes, suggesting that low concentrations of PGE_2_ cause hTSCs to undergo asymmetric differentiation. Endogenous PGE_2_ has also been shown to stimulate the proliferation of human MSCs [26], protect mouse embryonic stem cells from apoptosis through EP receptor activation [27], and enhance homing, survival, and proliferation of mouse and human hematopoietic stem cells that lead to increased numbers of repopulating cells and units [28]. As tendon-specific stem cells, TSCs play an important role in the repair of injured tendons by proliferating and differentiating *in vivo*. When tendons are injured, more tenocytes are needed, and TSCs must be activated to effectively repair injured tendons. Our results indicate that the constitutive baseline levels of PGE_2_, which are low, may be used to effectively expand TSCs for cell therapy of injured tendons by promoting proliferation and maintaining tendon homeostasis.

The beneficial effects of low PGE_2_ levels on TSCs have several potential applications in tendon tissue engineering. Since PGE_2_ at low levels can promote the stemness of TSCs, it may be used to maintain TSCs in culture. In addition, because low PGE_2_ levels can accelerate TSC proliferation, they could be used to quickly expand TSC populations for the use of cell therapy to treat injured tendons. Moreover, *in vivo* tendon injuries could be potentially treated by injecting low levels of PGE_2_ at the site of injury. This could enhance the healing of injured tendons because of the ability of low levels of PGE_2_ to stimulate self-renewal of TSCs and promote tenogenesis. A recent study showed that low levels of PGE_2_ injected into rat patellar tendons enhanced their structural properties (the ultimate load, stiffness, and elastic modulus) [29].

While this is the first study to demonstrate the biphasic effects of PGE_2_ on hTSCs, the molecular mechanisms responsible for these biphasic effects are yet to be investigated. PGE_2_ is known to exert its diverse biological effects through the EP receptors [14], [15], [30] and by differential activation of PKA types [13]. Hence, the biphasic response of hTSCs to PGE_2_ observed in this study may also involve multiple EP receptor subtypes and/or differential activation of PKA types. Also, while we have shown the beneficial effects of low PGE_2_ levels on hTSCs, one limitation of the study is the use of static culture without mechanical loading applied to hTSCs. However, tendons, and therefore the TSCs *in vivo*, are constantly subjected to mechanical loading, which regulates the expression levels of collagen type I, PPARγ, collagen type II, Sox9, and Runx2 genes. In addition, mechanical loading also increases PGE_2_ levels in both patellar and Achilles tendons [9], indicating a potential interaction between mechanical loading and PGE_2_. Additional studies are required to reveal the mechanisms behind this interaction. Further, we investigated only the long term effects (up to 6 days) of PGE_2_ treatment on hTSCs. It is known that exercise increases PGE_2_ levels in human blood only transiently, with maximum levels observed 2 hrs after exercise [31]. Therefore, it would be of interest to study the short term effects of PGE_2_ on hTSCs both *in vitro* and *in vivo.*

In summary, we showed in this study that PGE_2_ exerted biphasic effects on hTSCs: at low concentrations, PGE_2_ enhanced their proliferation and expression of stem cell markers, whereas high concentrations of PGE_2_ were detrimental to hTSCs, because they reduced their proliferation and induced non-tenocyte differentiation. These results suggest that, on one hand, low levels of PGE_2_ promote tendon homeostasis by maintaining hTSCs and tenogenesis; on the other hand, high levels of PGE_2_ in tendons may induce differentiation of hTSCs into non-tenocytes and thus lead to the development of the degenerative tendinopathy often seen in clinical settings.
